# The Effectiveness of Schema Therapy in Individuals Who Committed Crimes: A Systematic Review

**DOI:** 10.1177/15248380241254082

**Published:** 2024-05-21

**Authors:** Marta Sousa, Cláudia Gouveia, Olga Cunha, Andreia de Castro Rodrigues

**Affiliations:** 1 Psychology Research Center (CIPSI), School of Psychology, University of Minho, Campus de Gualtar, Braga; 2 Lusófona University, HEI-Lab: Digital Human-Environment Interaction Labs, Porto, Portugal; 3 William James Center for Research & ISPA, Lisbon, Portugal

**Keywords:** schema therapy, psychological intervention, systematic review, individuals who committed crimes, prison, community

## Abstract

Research suggests that individuals who commit crimes often exhibit various early maladaptive schemas (EMSs). EMSs are a broad and pervasive theme or pattern consisting of memories, emotions, cognitions, and bodily sensations concerning oneself and one’s relationships with others. Furthermore, EMSs play a crucial role in the onset and maintenance of different types of offending behaviors, highlighting the need to implement schema therapy (ST) for perpetrators. Therefore, the present systematic review assesses the effectiveness of ST for individuals who committed crimes. Four databases (PubMed, Scopus, Web of Science, and Scielo) were searched for studies examining the effectiveness of ST for individuals who committed crimes. Seventeen studies were identified, but only 15 met the criteria for inclusion. Results showed that ST can lead to beneficial effects in EMSs, schema modes, personality symptoms, and risk factors to commit crimes (e.g., cognitive distortions). However, the studies, besides being scarce, revealed some methodological limitations. ST is a promising therapy for individuals who committed crimes, despite the studies’ methodological shortcomings, which prevent us from drawing more firm conclusions. Although promising, more research is needed to enhance our understanding of the impact of ST therapies in forensic settings.

Perpetrators of crimes can cause much harm to the victims and society (Guay et al., 2019; Hailes et al., 2019). Therefore, it is crucial to develop appropriate and effective psychological interventions to mitigate the risk of these individuals causing harm to new victims. To date, cognitive-behavioral therapy (CBT) has been the predominant approach for treating various types of perpetrators (e.g., violent crimes, sexual violence, intimate partner violence [IPV]) (Butters et al., 2021; Gannon et al., 2019; Sousa et al., 2022). However, the empirical study of its effectiveness across different index offenses and settings has produced inconsistent results (e.g., Beaudry et al., 2021; Gannon et al., 2019; Sousa et al., 2022). Specifically, the effectiveness of most CBT-based treatments in prison settings is yet to be demonstrated conclusively (Beaudry et al., 2021; Byrne & Ghráda, 2019; Hopkin et al., 2018). The disparity in the outcomes arises from additional factors or variables that have not been considered in the treatment, such as personality disorders (PDs) (Bernstein et al., 2019). Studies indicate that PDs are highly prevalent in prison settings (e.g., Brazão, Motta, Rijo, Pinto-Gouveia et al., 2015; Mundt & Baranyi, 2020), highlighting their significant relevance to criminal behavior (Bernstein et al., 2019; Cavelti et al., 2021; Flórez et al., 2019; Fridell et al., 2008). Borderline PDs are prevalent in prison settings, affecting approximately half of the population, whereas antisocial PD is observed in over eighty percent of incarcerated individuals (Livanou et al., 2019; Mundt & Baranyi, 2020). Despite these high rates, CBT programs often focus on managing aggressive behavior and may not adequately address the symptoms, traits, and underlying structures associated with PDs (Bernstein et al., 2019).

Moreover, although studies indicate the positive impact of CBT programs on the mental health of perpetrators (Yoon et al., 2017), doubts have been raised regarding their sustained efficacy over the long term (Johnsen & Friborg, 2015). Consequently, some researchers propose that interventions should address deeper structures (i.e., early maladaptive schemas [EMSs]) to provide a longer-lasting change in the perpetrator’s cognitive functioning (Brazão et al., 2018a; M.Keulen-de Vos et al., 2013).

Cognitive information processing occurs across various levels of operation. Following Hollon and Kriss’s taxonomy (1984), these levels are classified into three types: structures (or schemas), processes, and cognitive products (Segal & Shaw, 1988). Schemas represent foundational cognitive structures in which all information is internally organized, serving as the basis for shaping perceptions about oneself, others, and the world (Beck, 1963). Cognitive processes involve the interaction between cognitive components, such as attention and codification, facilitating information processing. Guided by schemas, cognitive products, namely beliefs, emerge from these cognitive components. Consequently, addressing the perpetrators’ problematic cognitions requires altering cognitive processes and targeting underlying schemas (Gannon, 2016). However, intervention programs that address schemas are still scarce (Sousa et al., 2022).

Following this perspective, some authors suggest using schema therapy (ST) for forensic populations (Sousa et al., 2022). ST (Young et al., 2003) is an integrative therapeutic approach beyond traditional cognitive-behavioral treatments. The therapy incorporates elements from various therapies (i.e., CBT, attachment, Gestalt, object relations, constructivist, and psychoanalytic schools), particularly for patients considered challenging to treat, such as those with PDs or individuals facing multiple serious mental health problems (Young et al., 2003). Young suggests that these patients do not adapt well to CBT, partly because of their difficulty identifying, accessing, and changing their cognitions and emotions (Young et al., 2003).

Central to ST is the concept of EMSs and schema modes. EMSs are akin to the concept of schemas in the taxonomy of Hollon and Kriss (1984) but with a greater emphasis on their origin. Young defines EMSs as “a broad, pervasive theme or pattern, comprised of memories, emotions, cognitions, and bodily sensations, regarding oneself and one’s relationships with others” (Young et al., 2003, p. 7). These EMSs originate from early negative experiences with significant others and continue to evolve throughout the developmental process (Young et al., 2003). Maladaptation stems from the rigidity of these beliefs, which, when triggered, lead to incorrect interpretations and attributions of events, known as cognitive distortions (Brazão et al., 2017). In addition, schema modes are defined as the prevailing emotional states experienced by a person, which can be adaptive and nonadaptive (Young et al., 2003). The dysfunctional schema mode manifests when particular EMSs or coping responses give rise to distressing emotions, avoidance reactions, or self-destructive behaviors, assuming control over an individual’s functioning (Young et al., 2003). ST focuses on the exploration of the developmental origins of psychopathology, ingrained patterns of social and psychological functioning, as well as maladaptive cognitions and behaviors (Martin & Young, 2010).

Young et al. (2003) proposed 18 EMSs that could be clustered into five EMSs domains of unmet or violated core needs, including disconnection and rejection (in this field, the unfulfilled basic needs were safety, stability, and nurturance), impaired autonomy and performance (autonomy and competence), impaired limits (internal limits, responsibility toward others and long-term goal orientation), other-directedness (self-directedness), and overvigilance and inhibition (spontaneity and playfulness). Moreover, the authors differentiated 18 schema modes divided into four organizational domains: (a) child modes (i.e., referring to emotional responses that are nearly universal in children, such as sadness); (b) dysfunctional coping modes (i.e., which refers to the extreme attempt to deal with schema activation through surrender, avoidance, and overcompensation); (c) dysfunctional parent modes (i.e., referring to internalized harsh parental demands or punitive criticism), and the (d) healthy adult mode (i.e., healthy self-reflection and feelings of joy and pleasure). Perpetrators of crimes tend to exhibit different types of EMSs (Daffern et al., 2016; Horsley & Ireland, 2010; Oettingen et al., 2023), and researchers have stated that EMSs play an important role in the onset and maintenance of different types of offending behavior (Huang et al., 2023; M. E.Keulen-de Vos et al., 2016). As such, a positive association was found between mistrust/abuse, insufficient self-control, and entitlement EMSs with antisocial behavior in samples of adult perpetrators (Lavoie & Harwood, 2022; Gilbert & Daffern, 2013; Shorey et al., 2014). Furthermore, some EMSs have been associated with deficits that are risk factors for some types of offending (Janovsky et al., 2020; Sigre-Leirós et al., 2015).

## Current Study

A substantial body of research demonstrates the effectiveness of ST with non-forensic populations across various types of PDs (Taylor et al., 2017; Zhang et al., 2023). Additionally, research has underscored the need and utility of applying ST to forensic populations (e.g., Brazão, Motta, Rijo, Pinto-Gouveia et al., 2015; M.Keulen-de Vos et al., 2013; Sigre-Leirós et al., 2015). Therefore, the present study aimed to systematically review the current evidence on the effectiveness of ST (either alone or in combination with a guideline treatment) with justice-involved individuals within a forensic setting. Considering the impact of EMSs/schemas modes on the origin and maintenance of criminal behavior (Huang et al., 2023; M. E.Keulen-de Vos et al., 2016), we aim to assess their effectiveness in the short term (i.e., in reducing EMSs and symptoms, as well as criminogenic needs, i.e., risk factors for recidivism) and in the long term (i.e., in reducing recidivism rates).To our knowledge, this is the first review attempting to systematize this information, encompassing different types of perpetrators.

## Method

### Protocol and Registration

The systematic review protocol follows the Preferred Reporting Items for Systematic Reviews and Meta-Analysis (PRISMA) guidelines (Moher et al., 2010). This review was registered on OSF REGISTRIES (reference: 10.17605/OSF.IO/ZEF76).

### Eligibility Criteria

Studies meeting the following criteria were considered for inclusion: (a) empirical studies with quantitative methods (i.e., not literature reviews, theoretical articles, qualitative studies, and commentaries or letters to the editor). In the case of case studies, we only included them if they had quantitative data; (b) studies with male perpetrators of all types of crime serving a prison or community sentence; (c) studies that examined ST (or in combination with guideline treatment) (i.e., no restrictions were made about therapeutic format—individual or group/and in-, or outpatient setting); (d) studies that reported as outcomes changes in the population’s criminogenic needs, symptomatology and/or in EMSs and/or in reconviction rates; (e) studies written in English or Portuguese. No restrictions regarding the year of publication were made.

### Information Sources and Search Process

The subsequent equation was employed to ascertain the pertinent articles: (“Inmate*” OR “Incarcerat*” OR “Prison*” OR “Sentenced” OR “Detainee*” OR “Felon*” OR “Remand” OR “Criminal*”OR “Perpetrator” OR “Batterer” OR “Offender”) AND (“schema therap*” OR “schema group therap*” OR “schema mode therap*” OR “schema focused” OR “young’s model” OR “schema*”). In November 2023, two independent researchers with an MSc in Applied Psychology ran a search utilizing the specified equation. The search encompassed four electronic databases, exploring titles, abstracts, and keywords.: PubMed, Scopus, Web of Science, and Scielo. Moreover, the reference lists of several studies and specialized interdisciplinary journals were checked.

### Study Selection

Studies identified through equation search were imported into Rayyan software (Ouzzani et al., 2016), and the duplicates were deleted. Then, the first and second authors independently read the titles and abstracts, and the papers were selected for full-text analysis. Any discrepancies were resolved through discussion.

### Data Extraction

The same authors extracted data from the selected studies using an Excel standardized data extraction sheet. Data extraction included country of origin, year of publication, sample characteristics (i.e., treatment group, control group (if applicable), number of participants, age, gender, index offense), intervention characteristics (i.e., number of sessions and duration of treatment), setting, and pre-, post-, and follow-up treatment results regarding reported outcomes of interest, and completion rates. Following PRISMA recommendations, we calculated interrater agreement, which strongly agreed with Cohen’s Kappa coefficient (*K* = .89).

### Quality Assessment

The methodological quality of all included studies was assessed through the Mixed Methods Appraisal Tool (MMAT; Hong et al., 2018). The MMAT includes two screening questions (e.g., “Are there clear research questions?” and “Do the collected data allow to address the research questions?”) and five items to assess the methodological quality of studies, depending on the design of the study. Each item is classified as “yes,” “no,” or “don’t know.”

### Data Analysis

The employed method utilized a narrative synthesis approach, encompassing the presentation of narrative text and tables summarizing the data. This format enables readers to assess outcomes in the context of variations in study designs and potential sources of bias within each of the studies under review.

## Results

### Selection of Evidence Sources

**Figure 1.**
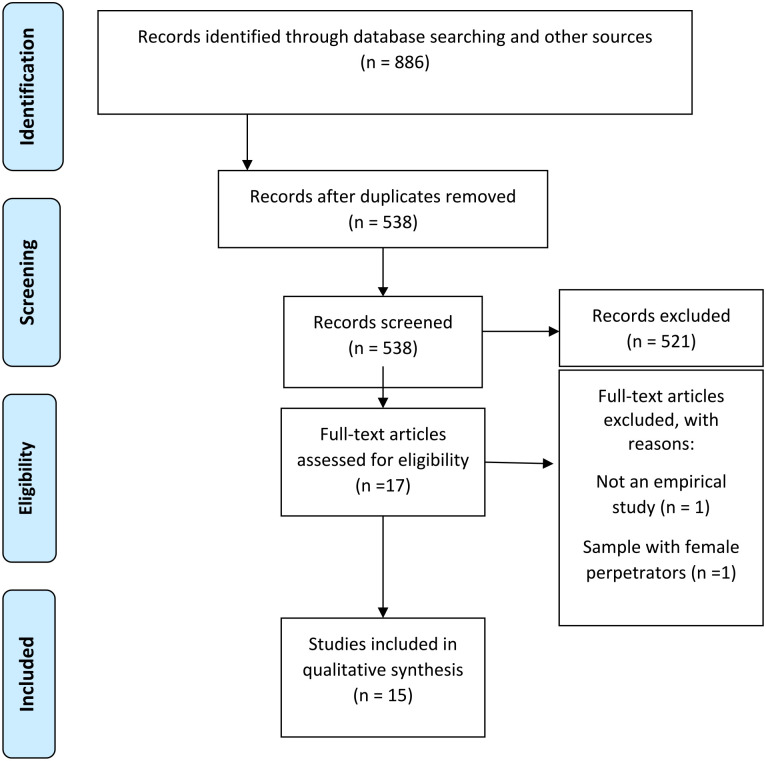
Preferred Reporting Items for Systematic Reviews and Meta-Analysis flow diagram of the study selection process.

A total of 886 studies were identified from the database search and manual reference checking. After removing duplicates, 538 were screened based on title and abstract. The eligibility of 17 studies was evaluated based on inclusion and exclusion criteria by reviewing their full texts. Ultimately, 15 studies satisfied all eligibility criteria for inclusion. Figure 1 provides a flowchart of the study selection process, including reasons for exclusion.

### Quality Assessment

Of the included studies, three studies showed all criteria of excellence (Brazão, Da Motta, Rijo, do Céu Salvador et al., 2015; Brazão, Da Motta, Rijo, Salvador et al., 2015; Chakhssi et al., 2014), six presented four out of five criteria of excellence (Bernstein et al., 2012, 2023; Brazão, Rijo, Salvador, Pinto-Gouveia et al., 2017, 2018a, 2018b, 2021), and three showed three out of five criteria (Capinha et al., 2023; de Klerk et al., 2022; Wilson et al., 2013). The remaining three studies did not show an acceptable quality and low risk of bias, with one study meeting only two criteria (Doyle et al., 2016) and two studies presenting only one criterion (Jalali et al., 2017, 2019).

### Study Characteristics

#### Sample

The sample size in the studies ranged from 1 to 254. From the studies, it was possible to observe that all samples were entirely composed of male individuals (*n* = 14; Bernstein et al., 2012, 2023; Brazão, Da Motta, Rijo, do Céu Salvador et al., 2015; Brazão, Da Motta, Rijo, Salvador et al., 2015; Brazão, Rijo, Salvador, Pinto-Gouveia et al., 2017, 2018a, 2018b, 2021; Capinha et al., 2023; Chakhssi et al., 2014; Doyle et al., 2016; Jalali et al., 2017, 2019; Wilson et al., 2013), except for one study, which predominantly had a male sample (de Klerk et al., 2022) (see Table 1).

**Table 1.** Characteristics of the Included Studies.

Study	Design	Sample Characteristics	Country	Treatment Program and Type	Intervention Format, Timing, and Setting	Control	Outcome Measures	Outcome/Dropout Rates
Bernstein et al. (2012)	RCT	*n* = 30 *I* = 16 *C* = 14	Netherlands	ST	Individual, 3 years, hospital	TAU	Clinical change (36 months follow-up)	ST patients showed more rapid progress and received both supervised and nonsupervised leave more quickly. But the differences did not reach a significant level. No information regarding rates of completion treatment.
Bernstein et al. (2023)	RCT	*n* = 103 *I* = 54 (*M* = 38.81; *SD* = 8.92) *C* = 49 (*M* = 36.45; *SD* = 10.77)	Netherlands	ST	Individual, 3 years, hospital	TAU	Clinical change (36 months follow-up)	ST was superior in both primary outcomes (i.e., attaining supervised and unsupervised leave and PD symptoms) and in some secondary outcomes (i.e., temperament, strengths and vulnerabilities, EMS, schema modes, psychopathology) than TAU. 25.9% of the participants did not complete the treatment.
Brazão, Da Motta, Rijo, do Céu Salvador et al. (2015), Brazão, Da Motta, Rijo, Salvador et al. (2015)	RCT	*n* = 48 *I* = 24 (*M* = 27.26; *SD* = 7.37) *C* = 24 (*M* = 29.50; *SD* = 5.83)	Portugal	ST-based therapy	Group, 40 sessions, prison	TAU	Clinical change (no follow-up)	ST patients had lower scores in Maladaptive Processes and on the majority of specific EMS (except in Mistrust/Abuse) when compared to the control group. ST patients had significantly lower scores at the end of treatment in anger trait (total score and subscales) and paranoia. No differences in anger-state and external shame between the groups. 11.11% of the participants did not complete the treatment.
Brazão, Rijo, Salvador, Pinto-Gouveia et al. (2017, 2018a, 2018b, 2021)	RCT	*n* = 254 *I* = 121 (*M* = 28.24; *SD* = 6.32) *C* = 133 (*M* = 28.74; *SD* = 6.14)	Portugal	ST-based therapy	Group, 40 sessions, prison	TAU	Clinical change (12 months follow-up)	Treatment participants presented a significant decrease in maladaptive processes, in EMS total score and all specific EMS, in external shame, paranoia, and emotional regulation in that control group. ST participants presented a significant increase in adaptive processes of anger control than control group. The improvements were sustained over time. The change over time in the outcome measures was not affected by personality pathology profiles. 14% of the participants did not complete the treatment.
Capinha et al. (2023)	Descriptive	*n* = 162 (*M* = 41.25; *SD* = 9.78)	Portugal	ST-based therapy + MI	Group or individual, 18 sessions, community	N/A	Recidivism rates (new arrest or conviction, 24 months after the end of the probation measure)	The recidivism rate for completers was 15.4%. 8% of the participants did not complete the treatment.
Chakhssi et al. (2014)	Single-case study	*n* = 1 (25 years old)	Netherlands	ST combined with movement therapy and milieu therapy	Individual, 4 years, hospital	N/A	Clinical change (24 months follow-up)	Significant decrease for four of the five EMS domains—Disconnection/Rejection, Impaired Autonomy/Performance, Impaired Limits and Overvigilance/Inhibition. Significant improvement in risk-related behaviors (i.e., social skills and Insight), and a decrease in risk of future violence and in psychopathy total score. The participant completed the treatment.
Doyle et al. (2016)	RCT	*n* = 63 *I* = 29 (*M* = 41.8; *SD* = 9.92) *C* = 34 (*M* = 42.74; *SD* = 12.44)	England	ST	Individual, 18 months (*M* sessões = 62), hospital	TAU	Clinical change (12 months follow-up)	No significant effects in posttreatment and follow-up on measures of anger, impulsiveness, and interpersonal style. Only significant differences in one EMS (defectiveness/shame) but in opposite expected direction. No information regarding rates of completion treatment.
Jalali et al. (2017)	Non-randomized control trial	*n* = 28 *I* = 14 (*M* = 31.87; *SD* = 4.95) *C* = 14 (*M* = 32.33; *SD* = 4.15)	Iran	ST	Group, 11 sessions, prison	NT	Clinical changes (no follow-up)	Significant increase in the experimental group in the level of self-esteem and in positive emotional regulation and a significant decrease in the level of negative emotion regulation compared to the control group. 23.07% did not complete the treatment.
Jalali et al. (2019)	RCT	*n* = 42 *I* = 21 (*M* = 32.60; *SD* = 4.95) *C* = 21 (*M* = 30.96; *SD* = 3.35)	Iran	ST	Group, 11 sessions, prison	NT	Clinical changes (no follow-up)	Significant improvement in depressive symptoms and EMS in the treatment group compared to the control group. All participants completed the treatment.
de Klerk et al. (2022)	Case-series study	*n* = 6 (*M* = 36; *SD* = 7.82; 83.33% were male)	Netherlands	ST	Group, 19 sessions*, hospital	N/A	Clinical changes (no follow-up)	Of the five male participants, three showed a significant decrease in maladaptive modes, and two showed no change in this dimension. In relation to healthy modes, four participants showed a significant increase, and one revealed no changes. One patient did not show significant changes in any of the psychopathology domains; one revealed a reliable change in interpersonal sensitivity, another in the level of hostility, another in hostility and sleep problems, and another reported significant changes in depressive symptoms. All participants completed the treatment.
Wilson et al. (2013)	Descriptive	*n* = 9 (*M* = 47.7)	New Zealand	ST-based therapy	Group and individual, 74 sessions, prison	N/A	Clinical change (no follow-up)	There is a significant decrease in cognitive distortions relating to denial, justification, and immaturity. The changes in emotional neediness, social sexual inadequacy, planning, and excitement/pleasure did not reach a significant level. All participants reduced their dynamic risk scores after treatment. 10% of the participants did not complete the treatment.

*Note.* C = Control; I = Intervention; N/A = Not Applicable; NT = Not therapy; RCT = Randomized Control Trial; ST = Schema therapy; TAU = Treatment as usual.

* The intervention has a second phase delivered according to a non-specific protocol, however, the results of this paper focus only on the effectiveness of the first phase of the program.

All studies focused on samples within the criminal justice context, including individuals in prison (*n* = 9; Brazão, Da Motta, Rijo, do Céu Salvador et al., 2015; Brazão, Da Motta, Rijo, Salvador et al., 2015; Brazão, Rijo, Salvador, Pinto-Gouveia et al., 2017, 2018a, 2018b, 2021; Jalali et al., 2017, 2019; Wilson et al., 2013), in forensic hospitals (*n* = 5; Bernstein et al., 2012, 2023; Chakhssi et al., 2014; Doyle et al., 2016; de Klerk et al., 2022) and in community (*n* = 1; Capinha et al., 2023). Regarding index offenses, two studies included patients with sexual offense convictions against women (Chakhssi et al., 2014; Wilson et al., 2013), one study included perpetrators of IPV (Capinha et al., 2023), and eight studies included perpetrators of different types of crimes (e.g., sexual offenses, murder, theft) (Bernstein et al., 2023; Brazão, Da Motta, Rijo, do Céu Salvador et al., 2015; Brazão, Da Motta, Rijo, Salvador et al., 2015; Brazão, Rijo, Salvador, Pinto-Gouveia et al., 2017, 2018a, 2018b, 2021; de Klerk et al., 2022). Lastly, four studies did not specify the type of crimes committed by the sample (Bernstein et al., 2012; Doyle et al., 2016; Jalali et al., 2017, 2019).

Seven studies were conducted in Portugal (Brazão, Da Motta, Rijo, do Céu Salvador et al., 2015; Brazão, Da Motta, Rijo, Salvador et al., 2015; Brazão, Rijo, Salvador, Pinto-Gouveia et al., 2017, 2018a, 2018b, 2021; Capinha et al., 2023), four studies in the Netherlands (Bernstein et al., 2012, 2023; de Klerk et al., 2022; Chakhssi et al., 2014), and two (18.18%) in Iran (Jalali et al., 2017, 2019). The remaining studies were managed in England (Doyle et al., 2016) and New Zealand (Wilson et al., 2012). Several study designs were identified, namely randomized controlled trials (RCT; *n* = 10, 66.67 %; Bernstein et al., 2012, 2023; Brazão, Da Motta, Rijo, do Céu Salvador et al., 2015, Brazão, Da Motta, Rijo, Salvador et al., 2015; Brazão, Rijo, Salvador, Pinto-Gouveia et al., 2017, 2018a, 2018b, 2021; Doyle et al., 2016; Jalali et al., 2017), descriptive studies (*n* = 2; 13.33%; Capinha et al., 2023; Wilson et al., 2013), non-RCT (*n* = 1, 6.67%; Jalali et al., 2017), a case-series design (*n* = 1; 6.67%; de Klerk et al., 2022), and a single-case study (*n* = 1; 6.67%; Chakhssi et al., 2014).

#### Treatment Approaches

Of the articles included, six studies used only ST (Bernstein et al., 2012, 2023; Doyle et al., 2016; Jalali et al., 2017, 2019; de Klerk et al., 2022), and nine studies included intervention programs strongly based on ST (Brazão, Da Motta, Rijo, do Céu Salvador et al., 2015; Brazão, Da Motta, Rijo, Salvador et al., 2015; Brazão, Rijo, Salvador, Pinto-Gouveia et al., 2017; 2018a, 2018b, 2021; Capinha et al., 2023; Chakhssi et al., 2014; Wilson et al., 2013). Nine studies included interventions delivered in group format (Brazão, Da Motta, Rijo, do Céu Salvador et al., 2015; Brazão, Da Motta, Rijo, Salvador et al., 2015; Brazão, Rijo, Salvador, Pinto-Gouveia et al., 2017; 2018a, 2018b, 2021; Jalali et al., 2017, 2019; de Klerk et al., 2022), four studies in individual format (Bernstein et al., 2012, 2023; Chakhssi et al., 2014; Doyle et al., 2016), and the remaining two in individual and group (Capinha et al., 2023; Wilson et al., 2013). The criteria for describing the duration of the intervention program vary across the studies: the studies focusing on the number of sessions indicated that the sessions vary from 11 (Jalali et al., 2017, 2019) to 74 sessions (Wilson et al., 2013); the studies specifying the time revealed that it varies from 17 months (Doyle et al., 2016) to 4 years (Chakhssi et al., 2014).

Fourteen studies used clinical change as an outcome, and one used recidivism (Capinha et al., 2023). The follow-up period ranged from 12 months to 36 months. Five studies had no follow-up. Moreover, the noncompletion rates varied between 0% and 25.9% (Bernstein et al., 2023). Two studies did not report these rates (Bernstein et al., 2012; Doyle et al., 2016).

#### Treatment Outcomes

Bernstein et al. (2012) conducted a comparative study to assess the effectiveness of ST in comparison to Treatment As Usual (TAU) for male forensic patients with PD. The ST intervention involved 3 years of twice-per-week individual sessions, while TAU was administered once per week. TAU is the treatment patients usually receive at each hospital: CBT, psychodynamic therapy, or client-centered therapy. Thirty participants were randomly assigned to the experimental group (*n* = 16) and the control group (*n* = 14). The authors used the resocialization variable (i.e., the gradual introduction of the person into the community with supervised and unsupervised leave) to measure effectiveness. As such, in Table 1, participants in the ST condition showed more rapid progress, attaining both supervised and unsupervised leave more quickly than the control group. However, it is important to note that these differences did not reach a statistically significant level. In a similar study, Bernstein et al. (2023) compared ST with TAU using a sample of individuals with PD and aggressive problems (*n* = 103; experimental group *n* = 54; control group *n* = 49). In this study, participants from the study carried out in 2012 were also included for analysis (Bernstein et al., 2012). The study utilized primary outcome variables, including rehabilitation measures (such as supervision and unsupervised leave) and PD symptoms, as well as secondary outcome variables, encompassing violence risk, institutional incidents, schema modes, and EMSs, to assess effectiveness. The results revealed that ST facilitated a faster progression of participants through rehabilitation compared to TAU, with a significant decrease in PD scores and temperament scale than the control group. Moreover, ST demonstrated a swiffer reduction in vulnerabilities and enhancement of strengths, which is advantageous over TAU in improving self-control and self-regulation. Further, participants in the ST condition showed a significant reduction in EMSs and schema mode than the control group.

Brazão and colleagues conducted several studies to test the GPS Program effects on psychological variables (Brazão, Da Motta, Rijo, do Céu Salvador et al., 2015; Brazão, Da Motta, Rijo, Salvador et al., 2015; Brazão, Rijo, Salvador, Pinto-Gouveia et al., 2017; 2018a, 2018b, 2021). GPS Program is grounded in a cognitive-interpersonal perspective, aiming to foster behavioral modification by promoting change in cognitive correlates (i.e., EMSs, cognitive distortions, and cognitive products). The two pilot studies (Brazão, Da Motta, Rijo, do Céu Salvador et al., 2015; Brazão, Da Motta, Rijo, Salvador et al., 2015) revealed that GPS effectively reduced maladaptive cognitive processes, specifically EMSs, anger-trait variable, and paranoia ideation. However, the program did not produce significant changes in anger-state and external shame. When considering clinical change analysis, no differences were found between groups on distribution categories for adaptive and maladaptive processes. However, significant differences were identified for total EMSs scores, emotional deprivation, defectiveness/shame, social isolation/alienation, and failure (Brazão, Da Motta, Rijo, Salvador et al., 2015). Moreover, differences emerged among groups regarding the distributions within clinical change categories for traits related to anger and its subscales, as well as paranoia (Brazão, Da Motta, Rijo, do Céu Salvador et al., 2015). Years later, with a larger sample size (*n* = 254), the authors concluded that not only did the GPS Program produce changes in posttreatment measures, but these changes were also maintained at a 12-month follow-up (Brazão, Rijo, Salvador, Pinto-Gouveia et al., 2017; 2018a, 2018b). In a secondary analysis of the results, the authors also concluded that the changes produced by the GPS Program were not significantly influenced by the PDs presented (Brazão et al., 2021).

Chakhssi et al. (2014) provided an overview of the 4-year therapeutic intervention based on ST for a 25-year-old male exhibiting psychopathic traits, including an assessment of the patient’s progress through recorded scores. The authors analyzed psychopathy, EMSs, behavioral, and risk measures. The results of this case study showed a significant decrease in most of the EMSs domains, risk of future violence, and psychopathy traits. Moreover, the patient showed a significant improvement in some risk-related behaviors, namely social skills and insight (i.e., insight about the nature of their problems), with no change in interpersonal hostility and psychical violence scores (Chakhssi et al., 2014).

In 2016, Doyle et al. conducted an RCT to evaluate the effect of ST on anger, impulsiveness, EMSs, and interpersonal style variables compared to TAU. In total, 63 participants were randomized to one of the two conditions (ST group = 29; TAU = 34), assessed at the baseline and then at 6, 12, 24, and 36 months on key dynamic outcome measures. ST therapy was delivered for at least 18 months, with 60-min sessions every week. The results showed no significant effects in posttreatment and follow-up on measures of anger, impulsiveness, and interpersonal style, with only significant differences in one EMSs. However, this difference was in the opposite direction expected, with participants in the ST group scoring higher on defectiveness/shame schema (Doyle et al., 2016).

In 2017 and 2019, Jalali et al. performed one semi-experimental study with a control group and one RCT to examine the effectiveness of ST on two different populations with specific needs. In both studies, ST was delivered in 90-min sessions each week. In 2017, the authors assessed the effectiveness of ST on self-esteem and emotion regulation in drug-addicted prisoners under the Methadone Maintenance Treatment (Jalali et al., 2017), concluding that ST produced more positive significant changes in variables in the study. In 2019, the focus was on 42 prisoners living with HIV and depression. The participants were randomly assigned to an experimental (*n* = 21) or waiting-list control group (*n* = 21) to evaluate the effectiveness of ST in reducing depression and EMSs. Participants in ST showed better scores in these two measures than in the control group (Jalali et al., 2019).

de Klerk et al. (2022) performed a case-series study (*n* = 6) with forensic patients with intellectual disability with two aims: to examine the effectiveness of ST in reducing maladaptive modes and psychological symptoms. The therapeutic intervention (ST) is structured in two phases: the initial phase involves a 19-session group protocol, while the subsequent phase occurs in a group format without a predefined protocol. Patient assessments were conducted at baseline and following the completion of the first treatment phase. The analysis of reliable changes demonstrated significant enhancements in emotional states and specific psychological complaints.

In 2013, Wilson et al. developed an intervention program for rape perpetrators based on the schemas and tested its effectiveness with an analysis of pre- and posttreatment measures (*n* = 10). Participants completed measures of personality psychopathology, risk, and dynamic sex offender risk factors. The results showed a significant decrease in cognitive distortions relating to denial, justification, and immaturity in participants, as well as in their risk scores. However, the changes in emotional neediness, social sexual inadequacy, planning, and excitement/pleasure did not reach a significant level.

Lastly, Capinha et al. (2023) tested the effectiveness of CONTIGO Program in Portugal for perpetrators of IPV based on recidivism rates. CONTIGO Program includes different phases and modalities: an initial individual motivational interview (MI) intervention, a structured group program inspired by ST, and a final individual MI intervention. The intervention program was delivered in an open-ended format with 90-min weekly sessions (Capinha et al., 2023). The term “recidivism” in this study refers to the occurrence of new charges related to IPV in police records. When completed by participants, the CONTIGO Program demonstrated a recidivism rate of 15.4%, which is lower than the average recidivism rate observed in comparable treatments (Capinha et al., 2023).

## Discussion

Over the last few years, interest in ST has increased as an alternative for individuals who have committed crimes to deal with PD symptoms, which are prevalent among these individuals, and to promote long-lasting changes in cognitive functioning. These individuals might present better results because ST addresses EMSs thought to be responsible for the onset and maintenance of offending behavior. With this systematic review, we aimed to provide an overview of effectiveness studies on ST as an intervention for individuals who have committed crimes. This study significantly contributes to the search for perpetrators’ rehabilitation, as it is, to the best of our knowledge, the first systematic review to provide a comprehensive perspective on intervention programs employing this therapeutic approach.

A total of 15 studies met the criteria for inclusion. Moreover, there was no significant variation in the number of interventions employing ST alone or in combination (six for ST alone and nine for ST in combination). The studies employing the ST by itself approach focused on reducing symptoms of PDs and psychopathology and promoting emotional regulation, which follows the primary targets of this therapy (Young et al., 2003). ST is an integrative therapy designed for patients considered difficult to treat, such as those with PDs or individuals with severe mental health challenges (Young et al., 2003). In contrast, the studies with a combined ST approach aimed at bringing about changes across various dimensions related to offending behavior to reduce the risk of recidivism. This finding follows the scientific literature that pointed out that EMSs are an element related to the offending behavior, though not the sole factor (e.g., Carvalho & Nobre, 2014; Sousa et al., 2022). Therefore, the intervention programs that aim to reduce the risk of recidivism also need to address specific criminogenic needs, such as cognitive distortions about women for perpetrators of rape (Wilson et al., 2013) or patriarchal beliefs and gender stereotypes for perpetrators of IPV (Capinha et al., 2023). Indeed, literature has demonstrated that interventions that follow the Risk-Responsivity-Need (RNR) Model (i.e., consider the risk, predictors of recidivism, and each person’s ability to respond) are more effective in reducing risk (Bonta & Andrews, 2017; Hanson et al., 2009). It implies that deciding on ST alone or in combination depends on the goals of the intervention program.

Consequently, most of the included studies utilized clinical change in the treatment targets to evaluate the effectiveness of intervention treatments. This approach deviated from the traditional method of assessing effectiveness (i.e., recidivism rates), as reported in the literature (Andersen & Skardhamar, 2017; Beaudry et al., 2021). The shift toward clinical changes can be substantiated in the intervention aims (i.e., some were not aimed at reducing recidivism) and by concerns surrounding the traditional method, particularly the absence of a universal definition for recidivism (Ostermann et al., 2015; Zgoba & Simon, 2005) and the underreporting of numerous crimes (Prentky et al., 1997). However, this tendency has been observed in previous reviews focused on evaluating the effectiveness of psychological interventions (e.g., Cunha et al., 2024; Sousa et al., 2022). In addition, most studies included individuals in prison or those undergoing mandated treatment in high-security hospitals. Only one study involved offenders serving sentences in the community, aligning with the therapy’s target population. As mentioned earlier, ST is often associated with populations deemed challenging to treat, exhibiting resistance to intervention, and experiencing severe mental disorders (Young et al., 2003) that are most prevalent in prison settings (Amirault & Beauregard, 2014; Cunha & Gonçalves, 2018; Sousa et al., 2024).

Overall, the current systematic review provides some evidence for the effectiveness of ST interventions with individuals who committed crimes regardless of the study design (quantitative randomized trials, quantitative non-randomized studies, quantitative descriptive studies, and qualitative studies), sample size, and type of offenses (e.g., rape, IPV). Positive outcomes have been found on psychological (e.g., PD symptoms, psychopathology, psychopathy, EMSs, schemas modes, emotional regulation, self-esteem, cognitive distortions, and risk of future violence) and behavioral variables (e.g., time to receive supervised and unsupervised leave, and recidivism). However, one study did not observe positive outcomes in the variables under study after the completion of treatment (Doyle et al., 2016). The poor quality of that work may explain the obtained result.

The effects of EMSs and schemas modes reduction can explain the positive results of ST. This reduction can be understood as a decrease in the prominence of these patterns in the individual’s self-concept (Young et al., 2003), allowing for a more realistic view of oneself, others, and the world while reducing cognitive distortions (Brazão et al., 2018a). Additionally, using experimental, cognitive, and behavioral strategies in this therapy provides an opportunity to enhance cognitive emotion regulation strategies (Jalali et al., 2017). Specifically, experimental techniques assist individuals in averting inefficient coping styles such as avoidance and excessive compensation (Fassbinder et al., 2016). Consequently, a decrease in cognitive distortions and ineffective emotion regulation strategies, among other factors, will influence the risk of violence and recidivism, as these constitute risk factors in criminal behavior (Bonta & Andrews, 2017).

Despite yielding positive results and having acceptable quality in most of the included studies, and with a prevalence of RCT design among them, drawing firm conclusions remains challenging due to some limitations. Specifically, most studies relied on small sample sizes, lacked follow-up assessments, or had a short follow-up period. Additionally, evaluating different intervention programs with distinct aims and psychometric measures makes direct comparisons between studies impossible. Moreover, the wide variation in the number of sessions or time in treatment raises questions about the “dose-effect” of ST in producing positive changes, and it also makes it impossible to compare results across studies (Olver, 2016). It is worth noting, however, that some of the highlighted limitations may stem from the exploratory nature of certain studies. Thus, even though there are 15 included studies evaluating the effectiveness of the intervention, six of them were carried out by the same research team in the same country (i.e., Portugal) and evaluated the same program.

### Strengths and Limitations

The present systematic review aimed to offer a comprehensive understanding of the empirical evidence of ST within a forensic setting involving individuals who have committed crimes. To the best of our knowledge, this work is the first to provide a comprehensive perspective on intervention programs employing this therapeutic approach in various forensic settings, such as prisons, forensic hospitals, and the community with adult men. Overall, our results allow us to conclude that ST showed promising outcomes, offering preliminary support for its use with individuals who have committed crimes (see Tables 2 and 3).

**Table 2.** Critical Findings.

Critical Findings
- ST for individuals involved with the justice system has a positive effect on EMS, schema modes, and personality symptoms. - ST for individuals involved with the justice system has a positive effect on reducing risk factors for committing crimes. - Assessing clinical changes is the predominant method for evaluating the effectiveness of intervention treatments. - Many studies involved individuals in prisons or those receiving mandated treatment in high-security hospitals. - The studies that aim to evaluate the effectiveness of ST intervention programs presented some methodological limitations.

*Note.* EMSs = early maladaptive schemas; ST = schema therapy.

**Table 3.** Summary of Implications for Practice, Policy, and Research.

Implications for Practice, Policy, and Research
1. Treatment programs should be undertaken within the framework of high-quality research, adhering to methodological principles that reduce bias, enabling us to draw solid conclusions. 2. Further research should include both self-report and official data to assess ST effectiveness on recidivism. 3. EMSs and schema modes should be accurately assessed during the evaluation phases to obtain a clear understanding of each perpetrator’s cognitive information processing. 4. There is some evidence of ST strategies in several variables of concern in crime prevention. 5. Efforts to target EMSs and schema modes should be integrated into intervention programs by incorporating ST, as it may facilitate more enduring changes and consequently promote well-being and reduce recidivism rates.

*Note.* EMSs = early maladaptive schemas; ST = schema therapy.

However, despite the contributions, some limitations should be mentioned. The first limitation identified was the absence of studies in languages other than English, which would allow a greater understanding of the use of ST with the target population in other countries. Moreover, the variability of the designs, treatment targets, and psychometric measures to assess clinical changes prevents us from making more firm conclusions regarding ST efficacy. Finally, the review’s exclusive focus on men prevents the results from being generalized to women who commit crimes. Future studies should address ST-based programs for women, analyzing their effectiveness.

## Conclusion

The main aim of the current systematic review was to provide an overview of effectiveness studies on ST as an intervention for individuals who have committed crimes. This study enables us to establish the positive effects of ST integration in psychological intervention on psychological variables (e.g., PD symptoms, psychopathology, psychopathy, EMSs, schema modes, emotional regulation, self-esteem, cognitive distortions, and risk of future violence), and behavioral variables (e.g., time to receive supervised and nonsupervised leave, and recidivism). Still, developing more studies analyzing ST with this specific population remains essential. Further research is essential to gain a deeper understanding of the impact of ST on the effectiveness of perpetrators’ rehabilitation, achieved through RCT with longer follow-up periods.
